# SARS-CoV-2 Infection in Pregnancy: Clinical Signs, Placental Pathology, and Neonatal Outcome—Implications for Clinical Care

**DOI:** 10.3389/fmed.2021.676870

**Published:** 2021-11-08

**Authors:** Sascia Moresi, Marco Dell'Aquila, Silvia Salvi, Roberta Rullo, Stefano Fruci, Francesca Stollagli, Vincenzo Arena, Antonio Lanzone

**Affiliations:** ^1^UOC di Ostetricia e Patologia Ostetrica, Dipartimento di Scienze della Salute della Donna e del Bambino e di Sanità Pubblica, Fondazione Policlinico Universitario A. Gemelli, IRCCS, Rome, Italy; ^2^UO Gineco-patologia e Patologia Mammaria, Dipartimento di Scienze della Salute della Donna e del Bambino e di Sanità Pubblica, Fondazione Policlinico Universitario A. Gemelli, IRCCS, Rome, Italy; ^3^Istituto di Clinica Ostetrica e Ginecologica, Università Cattolica del Sacro Cuore, Rome, Italy

**Keywords:** pregnancy, SARS-CoV-2, placenta, chronic histiocytic intervillositis, transplacental transmission of viruses

## Abstract

**Introduction:** The current COVID-19 pandemic has been associated with high rates of mortality and significant morbidity. Both the risk of infection for pregnant women and the risk of vertical transmission have been evaluated, and the presence of the SARS-CoV-2 virus has been demonstrated both in the placenta and in the amniochorionic membranes. However, the actual effects of this pathogen on pregnancy and on placental morphology are still unclear.

**Objective:** To describe histopathologic findings in the placentas of women with SARS-CoV-2 infection during pregnancy and their correlation with clinical signs and perinatal outcome.

**Methods:** Placental tissues from pregnant women with SARS-CoV-2 infection delivering between March 2020 and February 2021 were analyzed.

**Results:** One hundred six placentas from women with SARS-CoV-2 infection during pregnancy who delivered in Fondazione Policlinico A. Gemelli were examined. Most of them were asymptomatic. All neonates had available test results for SARS-CoV-2 and only one resulted positive. Placental tissues mainly showed signs of maternal vascular malperfusion and of placenta injury in terms of syncytial node increase (96.2%), villar agglutination (77.3%), neointimal hyperplasia (76.4%), excessive fibrin deposition (43.3%), and chorangiosis (35.8%). No significant differences in the frequency of the histopathological lesions were observed according to maternal symptoms.

**Conclusion:** Looking to placental tissues from SARS-CoV-2 positive women at the screening performed close to delivery, placental injuries could be detected without any correlation with fetal and neonatal outcomes. We hypothesize that short latency between SARS-CoV-2 infection and delivery is the main reason for these observations.

## Introduction

In December 2019, the severe acute respiratory syndrome coronavirus 2 (SARS-CoV-2) was reported in Wuhan, China (1) and had rapidly spread throughout the world until it was declared a Pandemic by the WHO on March 11, 2020.

Severe acute respiratory syndrome coronavirus 2 represents a highly pathogenic coronavirus like SARS-CoV-1 and Middle East Severe Respiratory Syndrome coronavirus (MERS-CoV), but, despite these similarities, less severe outcome of COVID-19 (coronavirus disease-year 2019) is found in pregnancy than SARS and MERS (2).

Although several studies reported about clinical outcomes of pregnant women with COVID-19 and their infants, concerns remain about the rate of vertical transmission, the placental involvement, and its clinical implications. Recently published data suggested the presence of SARS-CoV-2 in the placenta and in the amniochorionic membranes by microbiological swabs (3). The presence of the virus in placental tissues was further confirmed by electron microscopy techniques, immunohistochemistry, and hybridization techniques (4, 5). According to these observations, the transplacental transmission of SARS-CoV-2 is possible and may cause placental inflammation and neonatal viremia (6).

However, both the histopathological picture of the placenta in the course of the SARS-CoV-2 infection and the risks for the mother and for the newborn is still not completely clear.

Severe SARS-CoV-2 infection with the development of the COVID-19 syndrome may result in sustained hypoxia (7) and hypercoagulability with the development of ischemic changes, potentially implicated in pregnancy complications such as fetal growth restriction (FGR), pre-eclampsia, and stillbirth. Furthermore, like other known viruses, the SARS-CoV-2 virus may be responsible for fetal abnormalities [e.g., microcephaly in Zika virus infection (8)] or direct placental damage [e.g., villitis from cytomegalovirus (9)], leading to FGR or fetal demise. Although the pathophysiology of COVID-19 is not completely understood, there is emerging evidence that it can cause a severe systemic inflammatory response and may result in a hypercoagulable state with widespread microthrombi (10). Evidence of venous thromboembolism and microangiopathic disease in almost every organ system, including lungs, kidney, heart, and brain, has been identified both clinically and by autopsy examination (10, 11).

Sharps et al., in a recent large review of 50 studies, showed how different histopathological patterns including maternal vascular malperfusion (MVM), fetal vascular malperfusion (FVM), and signs of inflammation were identified with varying frequencies in placentas of pregnant women affected by SARS-CoV-2 infection. They concluded that the frequencies of these lesions remained uncertain as most studies reported a small number of cases, did not use control groups, and examination was not blinded to the clinical condition. Although the presence of lesions was reported in placentas from women with SARS-CoV-2 infection, the severity of lesions was not compared between women with and without the disease. Moreover, most reports found no evidence of viral infection detected in placental tissue in SARS-CoV-2 or related infections (12).

The aim of this study was to describe the histopathological findings in a large cohort of placentas from pregnant women that developed an infection from SARS-CoV-2 during pregnancy.

## Materials and Methods

### Study Population, Setting, and Data Collection

This is a retrospective single center study, conducted between March 2020 and February 2021 in Fondazione Policlinico Universitario Agostino Gemelli, IRCCS, Rome, Italy.

During all these months, pregnant women, regardless of the symptoms, performed a quantitative reverse transcription-PCR (RT-qPCR) SARS-CoV-2 test on nasopharyngeal swab at admission in the Hospital.

A series of 181 women with confirmed infection for SARS-CoV-2 during pregnancy were included. For the aim of this study, multiple pregnancies and patients where placenta histopathological examination was not available were excluded (*n* = 75).

For the purpose of data analysis, clinical informations were extracted from maternal medical records of 106 pregnant women and entered into a personal computer database. Maternal characteristics such as age, body mass index (BMI), smoking habit, gestational age at SARS-CoV-2 infection, clinical symptoms and signs of SARS-CoV-2 infection, and delivery data such as gestational age, parity, mode of delivery, and estimated blood loss (EBL) were evaluated.

Perinatal variables used were birthweight, birthweight centile, neonatal gender, Apgar score at 1st and at 5th minute, rate of respiratory support at birth, and admission to neonatal intensive care unit (NICU). Birthweight centile was calculated according to a national standard curve (13). We defined neonates less than the 10th centile of birth weight for gestational age as being small for gestational age (SGA) (13).

The placentas of all patients with positive SARS-CoV-2 testing were collected for macroscopic and microscopic histopathologic examination. Placental weights were measured, and placental weight centiles were calculated according to Almog et al. (14).

The main endpoint was to assess if potential effects of the viral pathogen could correlate with histopathological patterns of placental injury. Sub-groups analysis based on SARS-CoV-2 related maternal symptoms and on the diagnosis of pneumonia were performed. SARS-CoV-2 related symptoms included were: body temperature >37.5°C, dyspnea, cough, hyposmia, ageusia, and diarrheal. Pneumonia diagnosis was made by clinical evidence and diagnostic imaging in order to estimate lung involvement (ultrasound lung examination on pregnant symptomatic women and chest Rx or CT scan on women after delivery).

### Placental Examination

The placentas were sent to the Pathology laboratory for histopathological examination. Specimens were fixed in 10% buffered formalin.

The macroscopical examination and the resulting grossing of the placentas was performed according to an internal protocol in adherence to the Amsterdam placental workshop group consensus statement (15), and it included an extensive sampling that comprised at least two sections of the umbilical cord and of the membranes and up to six full thickness sections of the placental disk.

Fetal and maternal surfaces of the placental disk were cut into slices of about one cm of thickness to better detect eventual macroscopic lesions.

Afterward, sections were formalin fixed, paraffin embedded (FFPE), and subsequently stained with Hematoxylin and Eosin (H&E).

Histopathological findings were classified as fetal, maternal, or inflammatory coherently with their origin (16–18). Among those lesions defined as maternal vascular supply disorders, we described those vascular alterations that can be considered as part of maternal vascular maldevelopments like decidual arteriopathy and an increase in the intervillous fibrin. Specific vascular alterations related to maternal vascular disorders were an increase of the syncytial knots involving terminal villi, villous agglutination, intervillous fibrin, and villous infarcts. Fetal vascular supply disorders suggesting developmental disorders were chorioangioma, chorioangiosis, and delayed villous maturation.

Inflammatory lesions included acute chorioamnionitis and chronic histiocytic intervillositis. The histopathological examination of the placentas was made blind by two pathologists (Vincenzo Arena and Marco Dell'Aquila).

This study is included in a collaboration project approved by the Institutional review board of the Institute of Obstetrics and Gynecology, Catholic University of Sacred Heart, that provides the placental tissues investigation in case of materno-fetal diseases.

### Statistical Analysis

Statistical analysis was performed by using the Statistical Package for Social Science (SPSS) Version 20. The Kolmogorov-Smirnov and the Shapiro-Wilk tests were used to assess the normality of data distribution. Continuous variables, normally distributed, were expressed as mean ± standard deviation (SD) and categorical variables were displayed as frequencies. The statistical analysis Student's T-test or Mann Whitney, when appropriate, was used for comparing the means. Furthermore, the chi-square test was used to characterize the placental histopathological findings, aiming to compare the frequencies between the two groups (symptomatic and asymptomatic). *P* < 0.05 were considered as significant.

## Results

### Maternal Clinical Data, Pregnancy, and Neonatal Outcome

A total of 106 SARS-CoV-2 infected women with a singleton pregnancy who delivered in our Institution were included in the study.

Most of the study women (*n* = 60/106; 56.6%) were asymptomatic. The remaining 46 patients (*n* = 46/106; 43.4%) had mild COVID symptomatology including fever (*n* = 30/46; 65.2%), hyposmia and ageusia (*n* = 23/46; 50%), cough (*n* = 17/46; 36.9%), upper respiratory infection symptoms (*n* = 17/46; 36.9%), dyspnea (*n* = 7/46; 15.2%), arthralgia (*n* = 7/46; 15.2%), asthenia (*n* = 7/46; 15.2%), diarrhea and vomiting (*n* = 6/46; 13.0%), and were in the most severe cases interstitial pneumonia. The 11% of all women with SARS-CoV-2 infection and almost 30% of symptomatic women developed pneumonia confirmed with instrumental diagnosis as previously stated (*n* = 12, on total: 11.3%, on COVID-19 symptomatic women: 26.1%). Two-thirds of women with a diagnosis of pneumonia required non-invasive ventilation (*n* = 9/12; 75%). Regarding maternal treatment, among 46 symptomatic women, 24% received antibiotic treatment (*n* = 11/46; 23.9%) and nearly 37% antithrombotic prophylaxis with low molecular heparin. Hydroxychloroquine was used in four women (*n* = 4/46; 8.7%) and steroids were administered in six patients (*n* = 6/46; 13%). Remdesivir was used in three cases (*n* = 3/46; 6.5%). None of these women was admitted to the intensive care unit and none required extracorporeal membrane oxygenation. No maternal death was recorded. The mean length of maternal hospitalization in asymptomatic women was 5.9 ± 2.6 days whereas it was of 7.5 ± 4.3 days in symptomatic women (*p* = 0.023). Demographic and clinical characteristics were presented in Table 1. Maternal mean age was 32.63 years, their mean BMI was within the normal range, and most of these were non-smokers (*n* = 78, 73.6%). At the time of diagnosis of SARS-CoV-2 infection, most women were at term of pregnancy, so the mean gestational age at delivery in weeks was 39 weeks. The percentage of women delivering with a vaginal birth was 66% in comparison to 34% delivering with cesarean section (CS), which is in line with the CS rate of our Institution. Estimated blood loss at delivery was within the normal range.

**Table 1:** Demographic, clinical maternal characteristics, and neonatal outcome.

**Variable**	**All COVID-19 cases (*n* = 106)**
Age (years)	32.63 ± 5.73
BMI (kg/m^2^)	24.18 ± 4.21
Gestational age at COVID-19 positivity (week)	37.17 ± 5.09
Smoking habit	
- Yes	28 (26.4)
- No	78 (73.6)
Parity	
- Nulliparous	48 (45.3)
- Parous	58 (54.7)
Gestational age at delivery (week)	39.17 ± 1.37
Mode of delivery	
- Vaginal	70 (66)
- Cesarean	36 (34)
Estimated blood loss at delivery (ml)	367.45 ± 290.24
Birthweight (g)	3188.94 ± 438.85
Birthweight centile	43.54 ± 28.12
Small for gestational age, *n* (%)	10 (9.4)
Apgar score (1st)	8.82 ± 0.65
Apgar score (5th)	9.76 ± 0.49
Gender	
- Male	52 (49.1)
- Female	54 (50.9)
NICU admission	9 (8.5)
Respiratory support at birth	4 (3.8)

*Continuous variables are expressed as mean ± SD, categorical variables as numbers and percentages. Wk, weeks of pregnancy; BMI, body mass index; NICU, Neonatal Intensive Care Unit*.

All neonates born to mothers with SARS-CoV-2 infection had available test results for SARS-CoV-2 *via* nasopharyngeal PCR at 24 h of age and only one of these had a positive test (1:106, 0.9%). No cases of neonatal death and fetal intrauterine death occurred. Among neonates born to infected mothers, four of them (3.8%) required respiratory support and nine (8.5 %) were admitted to NICU. The rate of admission to NICU of these full-term neonates could not be compared with the term “neonates general population rate” since in the first months of the COVID-19 pandemic, main neonates were admitted only for isolation in the intensive care unit. The majority of newborns had an appropriate weight for gestational age and normal range of Apgar score both at the first and fifth minutes from delivery. The SGA rate was 9.4%, the same as the general population.

### Placental Histopathological Findings Following SARS-CoV-2 Infection

In Table 2, placental histopathological findings of all placental tissues and according to COVID-19 symptomatology were reported. No significant differences were found between the two subgroups about lesions examined, placental weight, and placental weight percentile.

**Table 2:** Placental histopathological findings of all COVID-19 placental tissues according to COVID-19 symptomatology and diagnosis of pneumonia.

**Variables**	**All COVID-19 cases (*n* = 106)**	**COVID-19 symptomatic (*n* = 46)**	**COVID-19 asymptomatic (*n* = 60)**	***P-value* ^a^**	**COVID-19 symptomatic with pneumonia (*n* = 12)**	**COVID-19 symptomatic without pneumonia (*n* = 34)**	***P*-value^b^**
Placental weight (gr)	513.84 ± 102.76	525.24 ± 108.59	504.86 ± 98.03	0.307	512.58 ± 117.28	530.12 ± 106.82	0.636
Placental weight centile	19.18 ± 19.05	21.48 ± 20.11	17.42 ± 18.16	0.279	19.50 ± 14.26	22.18 ± 21.95	0.697
Thrombosis, *n* (%)	0	0	0	n.a.	0	0	n.a.
Accelerated maturation, *n* (%)	6 (5.7)	1 (2.2)	5 (8.3)	0.190	1 (8.3)	0 (0)	0.089
Chronic Histiocytic Intervillositis, *n* (%)	8 (7.5)	3 (6.5)	5 (8.3)	0.726	2 (16.7)	1 (2.9)	0.098
Infarct lesions, *n* (%)	14 (13.2)	5 (10.9)	9 (15)	0.534	2 (16.7)	3 (8.8)	0.453
Inflammatory lesions, *n* (%)	16 (15.1)	7 (15.2)	9 (15)	0.975	3 (25)	4 (11.8)	0.272
Delayed maturation, *n* (%)	16 (15.1)	6 (13.0)	10 (16.7)	0.784	0 (0)	6 (17.6)	0.119
Intervillar thrombosis, *n* (%)	18 (16.9)	6 (13.0)	12 (20)	0.344	0 (0)	6 (17.6)	0.119
Fibrotic villous, *n* (%)	26 (24.5)	13 (28.3)	13 (21.7)	0.434	1 (8.3)	12 (35.3)	0.075
Villar hypoplasia, *n* (%)	27 (25.5)	11 (23.9)	16 (26.7)	0.747	3 (25)	8 (23.5)	0.918
Chorangiosis, *n* (%)	38 (35.8)	19 (41.3)	19 (31.7)	0.305	5 (41.7)	12 (41.2)	0.976
Excessive fibrin deposition, *n* (%)	46 (43.4)	20 (43.5)	26 (43.3)	0.988	6 (50)	14 (41.2)	0.596
Neointimal hyperplasia, *n* (%)	81 (76.4)	38 (82.6)	43 (71.7)	0.188	7 (58.3)	31 (91.2)	0.010*
Villar Agglutination, *n* (%)	82 (77.3)	35 (76.1)	47 (78.3)	0.784	7 (58.3)	28 (82.4)	0.094
Syncytial node increase, *n* (%)	102 (96.2)	44 (95.6)	58 (96.7)	0.786	11 (91.7)	33 (97.1)	0.431

a *Comparison between symptomatic and asymptomatic cases*.

b *Comparison between symptomatic with and without pneumonia cases*.

*Continuous variables are expressed as mean ± standard deviation; categorical variables as number and percentages*.

*Placental weight centiles calculated according to Almog et al. (14). Histopathological findings were reported according to Benton et al. (18)*.

*The value is in bold since it is the only statistically significant value*.

The value indicated with * is the only statistically significant value.

Then, histopathologic findings between women with a diagnosis of SARS-CoV-2 related and unrelated pneumonia were described. No significant differences were found between the two subgroups regarding placental weight, placental weight centile, and most of the lesions examined. We observed a significative higher rate of neointimal hyperplasia in women without a diagnosis of SARS-CoV-2 related pneumonia with respect to women with interstitial pneumonia (*p* = 0.010).

**Figure 1:**
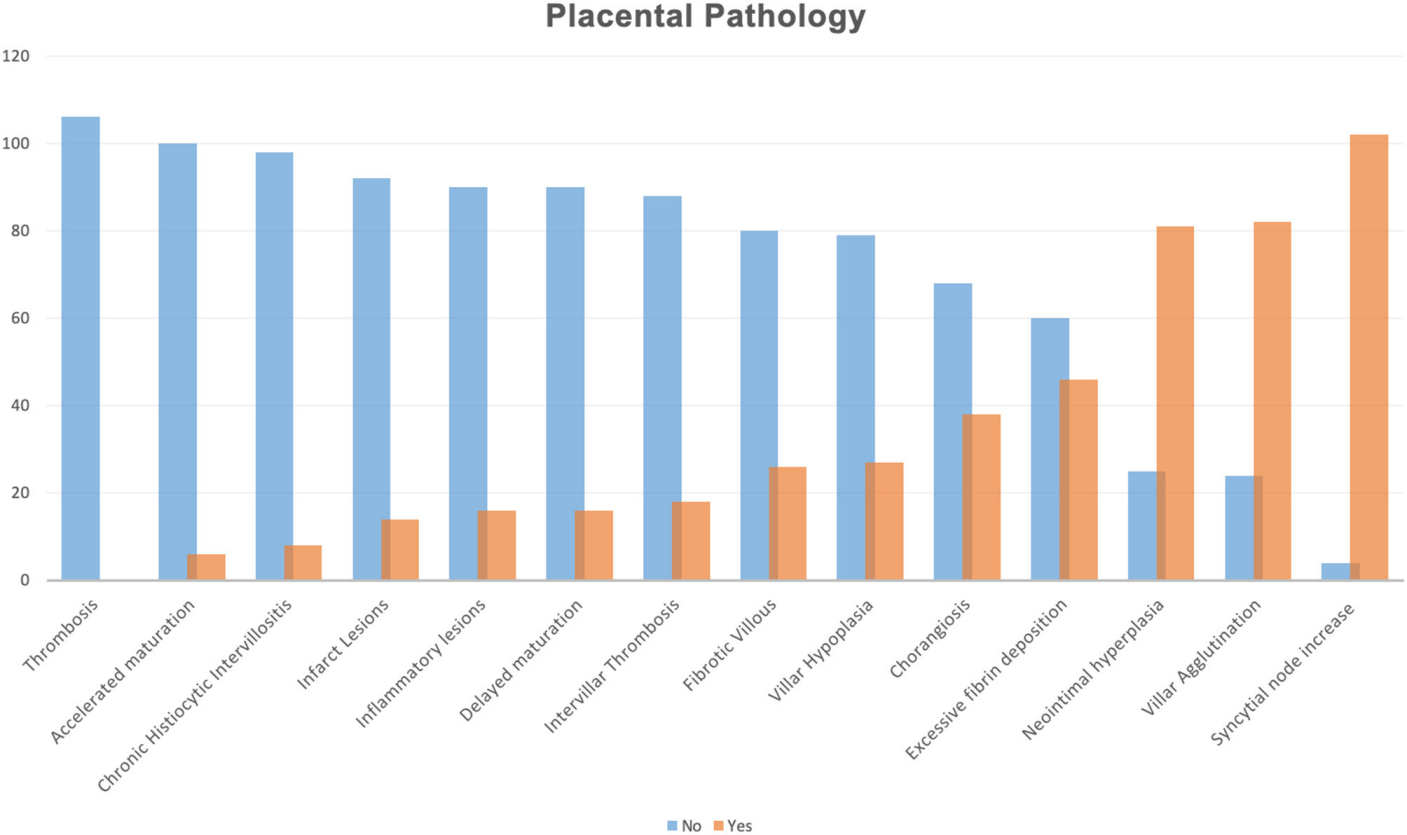
Histopathological lesions illustrated according to each frequency in all placental samples. Histopathological findings were reported according to Benton et al. (18).

All histopathological lesions were also illustrated according to each frequency in Figure 1. We described the presence of high frequency in our population of syncytial node increase (96.2%), villar agglutination (77.3%), neointimal hyperplasia (76.4%), excessive fibrin deposition (43.3%), and chorangiosis (35.8%).

**Figure 2:**
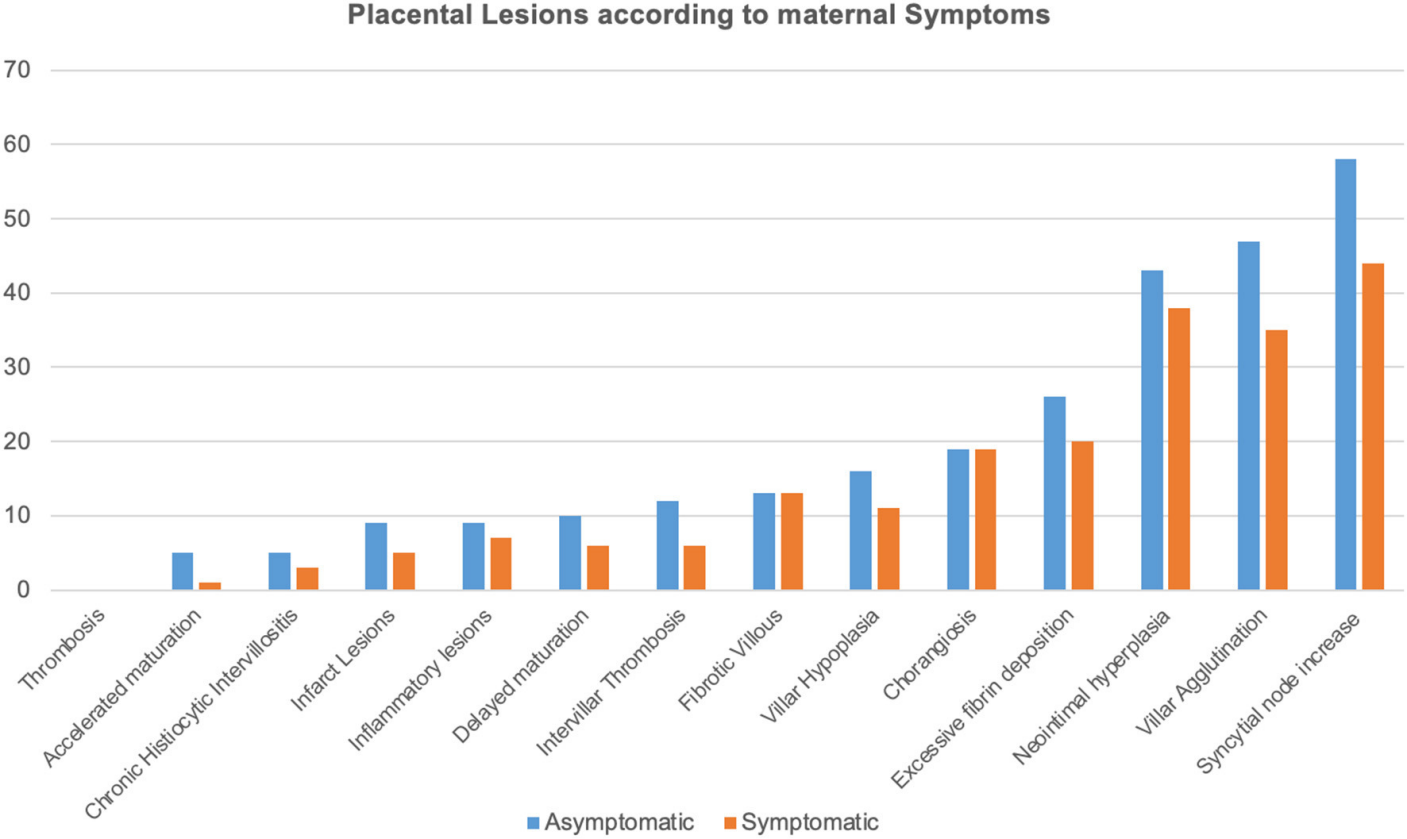
Histopathological lesions are illustrated according to each frequency and to maternal symptoms as SARS-CoV-2 related symptoms included body temperature >37.5°C, dyspnea, cough, hyposmia, ageusia, and diarrhea. Histopathological findings were reported according to Benton et al. (18).

No significant differences in the frequency of the histopathological lesions were observed according to maternal symptoms (Figure 2).

**Figure 3:**
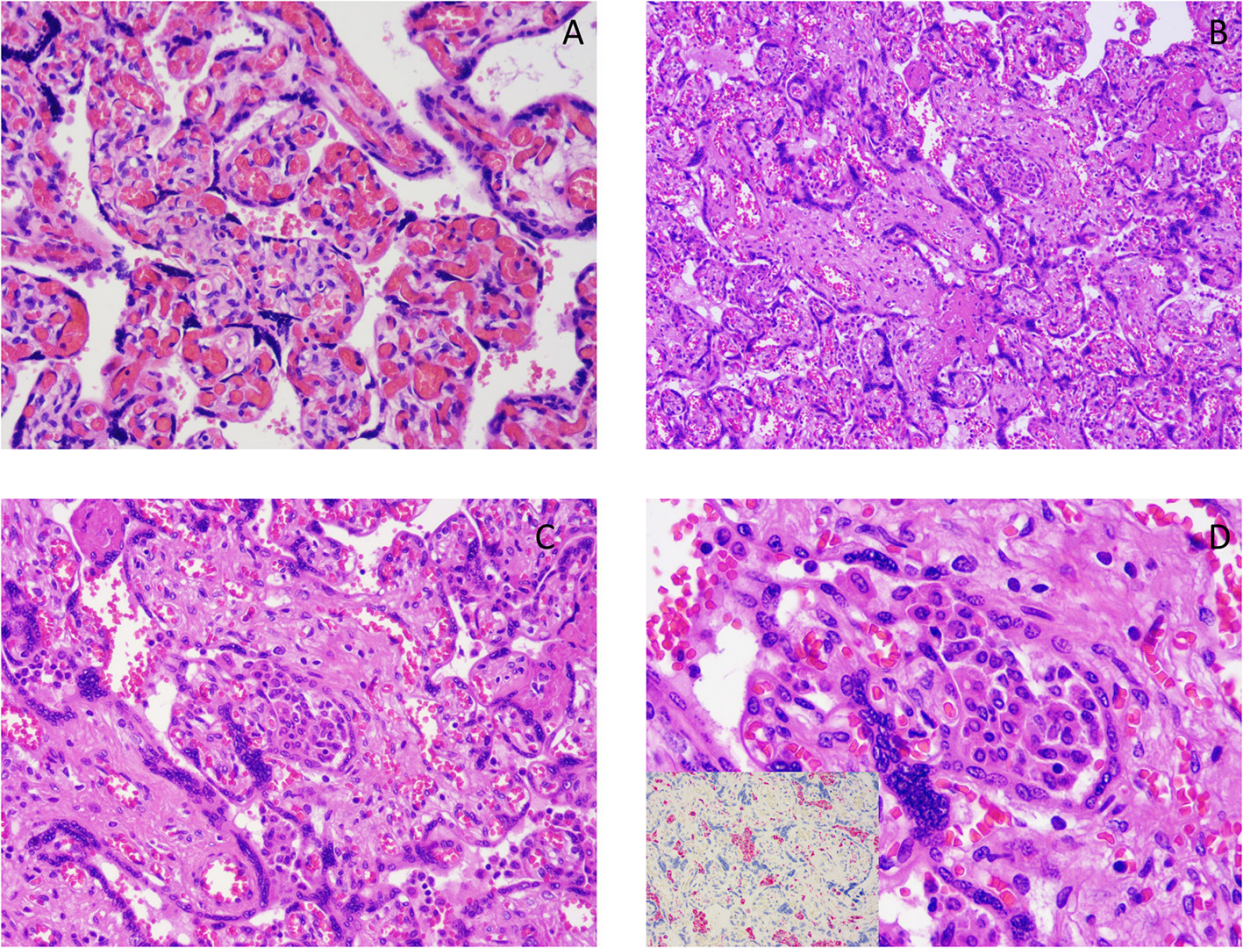
**(A)** Chorangiosis. **(B–D)** Chronic histiocytic intervillositis images showing a histiocytic infiltrate in the intervillous space. Pictures at 10× **(B)**, 20× **(C)**, 40× **(D)**. The image, inserted in the corner of **(D)** shows the results of immunohistochemistry for CD68 (red chromogen), a monocyte lineage marker, highlighting the histiocytic nature of the infiltrate.

Our series was characterized by a high prevalence of chronic histiocytic intervillositis (CHI) (*n* = 8, 7.5%). Pathology of chronic histiocytic intervillositis and fetal villi with chorangiosis are illustrated in Figure 3.

## Discussion

More than a year has passed since the beginning of the COVID-19 pandemic and significant advances were made in understanding the effects of the infection in pregnant women and their infants, but there still remains to investigate.

At the beginning of the outbreak of the infection in Italy, our center was designed as a referral COVID-19 maternity hospital, allowing us to include in this study a total of 106 placentas of pregnant women with SARS-CoV-2 infection, the largest sample described in the literature until now.

The majority of these women delivered at term with the prevalence of vaginal delivery (66%). According to other published data (19, 20), no evidence of clear benefits in performing CS in women SARS-CoV-2 infection has been demonstrated.

No major adverse maternal and perinatal outcomes were detected. These findings may be explained by the timing of the infection, the duration, and the severity of maternal symptoms. Most of our patients were discovered to be positive at the term of pregnancy for the universal screening for SARS-CoV-2 performed at the admission for labor and delivery.

More than half were asymptomatic (60/106: 56.6%) and among the symptomatic women (46/106: 43.4%), 26% (12/46: 26.1%) had pneumonia. None of these women had the most severe form of the disease and none was admitted to the intensive care unit. The majority of newborns had an appropriate weight for gestational age and a normal range of Apgar score.

In relation to the placental examination, our cohort was characterized by a mean placental weight centile of 19.2, assessed according to Almog et al. (14). No statistically significant difference was found according to SARS-CoV-2 related maternal symptoms or pneumonia. Placental tissues mainly showed signs of MVM, in line with what was already reported (5, 21–23). We described the presence of placenta injury in our population in terms of syncytial node increase (96.2%), villar agglutination (77.3%), neointimal hyperplasia (76.4%), excessive fibrin deposition (43.3%), and chorangiosis (35.8%). Although we did not find specific histopathological findings related to the SARS-CoV-2 infection, the high prevalence of MVM and chorioangiosis suggest a potential role of possible low efficiency of oxygen transfer between the intervillous space and the fetal circulation.

However, no differences were observed between symptomatic and asymptomatic women, nor even considering the diagnosis of pneumonia.

According to literature (21), these changes may also reflect a systemic inflammatory or hypercoagulable state, even if in our sample we observe no evidence of placental thrombosis (0%) and a low prevalence of inflammatory lesions (15%).

These placental histopathological findings did not influence pregnancy outcome, supporting the important role of the placenta as a defensive biologic filter for the fetus.

In our series, we found a shorter interval from the infection to the delivery: the gestational age at the COVID-19 infection diagnosis was closer to the term. Maybe in this brief period, the virus was not able to produce significant placental damage, thus affecting both obstetrics and neonatal outcomes. Most of the placentas of women with the most severe respiratory syndrome SARS-CoV-2 related that required hospitalization during the gestation was missing in our cohort as the majority of them, after healing from COVID-19, have chosen to deliver in their local hospitals. For these reasons, pregnant women who had the disease earlier in pregnancy are not included and since the antibody screening is not routinely performed in our institution, the correct dating of the maternal infection was not applicable. These are the main limitations of the study.

Although the microbiological examination of the placenta was not available, we found a low risk of vertical transmission. All newborns were tested at birth and after the third day of life and only one infant tested at birth resulted positive at the first swab for SARS-CoV-2. However, the virus did not seem to impact perinatal outcomes.

In this case of neonatal positivity, the mother had mild symptomatic SARS-CoV-2 infection (only fever) 7 days before delivery. She had a spontaneous vaginal delivery at 39 weeks of gestation and the mother was still positive during labor. The birth weight was 2,900 g (15° centile) (13) the Apgar score at first and fifth minutes was 9–10. Placental examination showed MVM (syncytial node increase, villar agglutination), FVF (neointimal hyperplasia, villar hypoplasia), and delayed villar maturation.

In literature, there is limited evidence to support the possibility of transplacental transmission.

The SARS-CoV-2 infection is mainly mediated by the binding of viral spike (S) protein to its corresponding receptor on the surface of the target cell which was supposed to be Angiotensin converting enzyme 2 (ACE2). A recent study revealed no expression of its receptor on the placenta surface (24, 25).

Finally, an interesting result that our case series highlights are the presence of chronic histiocytic intervillositis (CHI) in 8 out of 106 cases (7.5%). CHI is a rare entity with an estimated prevalence spanning between 6 out of 10,000 and 0.03–0.5% depending on the cohorts analyzed in second and third-trimester placentas (26–28). The association between CHI in the placenta and the SARS-CoV-2 infection has already been described in the literature (4, 21, 22). Even though we cannot define CHI as the hallmark histopathological finding in SARS-CoV-2 placental infection, its relatively high incidence in our case series is a relevant finding, especially when considering that the etiopathogenesis of this pathological picture is still not completely clear. Most CHIs tend to occur sporadically without a clear causative factor, but nonetheless, it has been associated with preterm deliveries, intra-uterine growth retardations, in utero deaths (29, 30), and with a very high risk of recurrences (31).

In conclusion, looking to placental tissues from SARS-CoV-2 positive women at the screening performed close to delivery, placental injuries in terms of syncytial node increase (96.2%), villar agglutination (77.3%), neointimal hyperplasia (76.4%), and excessive fibrin deposition (43.3%) could be detected although any correlations with fetal and neonatal outcome in terms of neonatal weight and centile, Apgar score, and NICU admission were founded. We hypothesize that short latency between SARS-CoV-2 infection and delivery is the main reason for these observations.

Although our observations are not conclusive due to the presence of the limitations of the study, we believe that this work, with its large case study, may represent a valuable basis that can contribute to our comprehension of the effect of the SARS-CoV-2 virus on pregnancy and in the placental physiopathology.

## Data Availability Statement

The raw data supporting the conclusions of this article will be made available by the authors, without undue reservation.

## Author Contributions

SM, MD, SS, RR, SF, FS, VA, and AL: contributed to conception and design of the study. SM, MD, RR, SF, and FS: organized the database. SS: performed the statistical analysis. SM, MD, RR, SF, FS, and SS: wrote sections of the manuscript. All authors contributed to manuscript revision, read, and approved the submitted version.

## Conflict of Interest

The authors declare that the research was conducted in the absence of any commercial or financial relationships that could be construed as a potential conflict of interest.

## Publisher's Note

All claims expressed in this article are solely those of the authors and do not necessarily represent those of their affiliated organizations, or those of the publisher, the editors and the reviewers. Any product that may be evaluated in this article, or claim that may be made by its manufacturer, is not guaranteed or endorsed by the publisher.
